# Dendritic cell vaccine treatment for indolent B cell non-hodgkin lymphoma: clinical trial in progress

**DOI:** 10.1186/2051-1426-2-S3-P76

**Published:** 2014-11-06

**Authors:** Yi Lin, Thomas Atwell, Adam Weisbrod, Mary Maas, Adam Armstrong, Michael Deeds, Peggy Bulur, Michael Gustafson, Zhe Zhang, Louis Porrata, Svetomir Markovic, Patrick Johnston, Ivana Micallef, David Inwards, Joseph Colgan, Stephen Ansell, Dennis Gastineau, Allan Dietz, Thomas Witzig

**Affiliations:** 1 Mayo Clinic, Rochester, MN, USA

## Introduction

We present the preliminary results of our clinical trial testing 2 vaccine strategies in patients with indolent B cell non-Hodgkin lymphoma (NHL; NCT01239875, http://clinicaltrials.gov). The primary objective is to determine the safety and feasibility of the vaccine approaches and secondary objectives are to describe clinical responses and identify corresponding immune changes.

## Methods

Autologous mature dendritic cells (mDC) were manufactured from leukapheresed cells of NHL patients. For patients with tumor lymph nodes deemed amenable to cryoablation by interventional radiologist (arm A), they received cryoablation of a node and injection of mDC into the cryoablated node followed by another 1 to 7 intratumoral mDC injections. Remaining patients had a tumor excised to generate tumor lysate ex vivo. mDC were pulsed with tumor lysate during DC maturation (arm B; DC-TL). The DC-TL vaccines were injected intradermally for 4 to 8 doses. Patients are monitored for one year after vaccines for adverse events and systemic tumor response. Correlative studies include cellular immune phenotype of peripheral blood and T cell intracellular cytokine productions. Planned accrual is 10 patients per arm (total = 20).

## Results

To date, 10 patients have accrued to arm A and 5 patients to arm B. All patients tolerated vaccine treatments without major adverse events. Of the 10 evaluable patients, there were 1 CR (arm B: 1 / 4; total: 1/10) and 4 PR (arm A: 3/6; arm B: 1 / 4; total: 5/10) for an ORR of 50% for both arms (Table 1). Responses have been durable for at least 1 year. Correlative studies suggest that immune changes can be used as prognostic biomarkers to predict response. Upon stimulation, responders' T cells had increased IFN-γ and or IL-17a and lower IL-4 production than non-responder T cells. Preliminary analysis of >80 immune phenotypes using flow cytometry and hierarchical clustering suggest that, after vaccination, many components within the immune system of responders change in a different manner from the non-responders.

**Table 1:** Patient responses

Arm	ID	Age/ Gender	Histology	Stage	FLIPI/IPI	# of prior Tx	DC doses	Best response	Time to next treatment or event (months)
A	LSA1	57M	Follicular	IVA	2	1	2	SD	25
A	LSA2	56F	Follicular	IIIB	4	2	2	SD	14
A	LSA3	69M	Follicular	IVA	3	4	2	PR	22
A	LSA4	60F	Follicular	IVA	3	3	2	PR	20
A	LSA5	64F	Follicular	IVA	5	8	8	PR	Not reached (12 mo at the time of abstract.)
A	LSA6	81M	Follicular	IVA	4	1	8	SD	Not reached (9 mo at the time of abstract.)
B	LSB1	60M	Follicular	IIIA	3	1	4	SD	6.5
B	LSB2	62F	Marginal zone	IVAE	4	6	4	SD	7.6
B	LSB3	65F	Follicular	IVA	3	2	8	CR	Not reached (13 mo at the time of abstract.)
B	LSB4	31F	Follicular	IIIA	2	0	8	PR	Not reached (12 mo at the time of abstract.)

## Conclusions

Both cryoablation and intratumoral mDC vaccination are feasible and safe in NHL. Treatment responses may correlate with immune system changes. Biosystems analysis method can be used to develop novel assays as predictive biomarkers of treatment response.

